# The mammalian neocortex new pyramidal neuron: a new conception

**DOI:** 10.3389/fnana.2013.00051

**Published:** 2014-01-06

**Authors:** Miguel Marín-Padilla

**Affiliations:** Department of Pathology and Pediatrics, The Geisel School of Medicine at DartmouthHanover, NH USA

**Keywords:** mammals, motor cortex, new pyramidal neuron, development, cortical layering, human

## Abstract

The new cerebral cortex (neocortex) and the new type of pyramidal neuron are mammalian innovations that have evolved for operating their increasing motor capabilities while essentially using analogous anatomical and neural makeups. The human neocortex starts to develop in 6-week-old embryos with the establishment of a primordial cortical organization, which resembles the primitive cortices of amphibian and reptiles. From the 8th to the 15th week of age, new pyramidal neurons, of ependymal origin, are progressively incorporated within this primordial cortex forming a cellular plate that divides its components into those above it (neocortex first layer) and those below it (neocortex subplate zone). From the 16th week of age to birth and postnatally, the new pyramidal neurons continue to elongate functionally their apical dendrite by adding synaptic membrane to incorporate the needed sensory information for operating its developing motor activities. The new pyramidal neuron’ distinguishing feature is the capacity of elongating anatomically and functionally its apical dendrite (its main receptive surface) without losing its original attachment to first layer or the location of its soma and, hence, retaining its essential nature. The number of pyramidal cell functional strata established in the motor cortex increases and reflects each mammalian species motor capabilities: the hedgehog needs two pyramidal cell functional strata to carry out all its motor activities, the mouse 3, cat 4, primates 5 and humans 6. The presence of six pyramidal cell functional strata distinguish the human motor cortex from that of others primates. Homo sapiens represent a new evolutionary stage that have transformed his primate brain for operating his unique motor capabilities, such as speaking, writing, painting, sculpturing and thinking as a premotor activity. Words used in language are the motor expression of thoughts and represent sounds produced by maneuvering the column of expiratory air by coordinated motor quivering as it passes through the larynx, pharynx, mouth, tongue, and lips. Homo sapiens cerebrum has developed new motor centers to communicate mental thoughts (and/or intention) through motor actions.

## INTRODUCTION

The vertebrate brain (including that of non-mammals), in the opinion of many, is a premotor organ that operates animals’ motor activities (Sperry, 1952; Ortega y Gasset, 1957; Llinás, 2001; Wolpert and Flanagan, 2010; Marín-Padilla, 2011). The brain’s motor neurons operate the animal musculature in the search for food and a mate and in evading danger. Humans, in addition, are capable of expressing thoughts (intentions) through motor actions. Words (spoken, written, sang, or in sing language) are simply motor expressions of thoughts. The column of expiratory air is maneuvered to produce sounds by synchronized muscular quivering as it passes through the larynx, pharynx, mouth, tongue, and lips. Ortega y Gasset (1957) expressed this idea best; “Therefore, there is no genuine motor activity without a previous thought, and there is no genuine thought if is not duly referred to a motor activity and enhanced by its relation with it”. Writing, painting, sculpturing, playing musical instruments and/or practicing sports are likewise coordinated motor activities for expressing mental thoughts or intentions.

The development of Broca’s area as well as some anatomical modifications in the larynx have been associated with the evolution human language. In my opinion, these transformations are not the cause but the consequences of the human need for expressing his thoughts through motor actions and the capability for accomplishing it. Humans could equally use their hands (even the feet) motor capabilities to express and communicate thoughts. Knowing that a cortical lesion in specific motor regions eliminate some motor activities tell us nothing about how the destroyed neurons were able of operating them. What type of cortical neuron, shared by all mammals, operates these coordinated and complex motor activities and how it could accomplish the task remain essentially unknown because it has not been adequately studied.

While our understanding of the synapse ultrastructure and biochemistry of synaptic transmission (the micro scale) and the interpretation of images from magnetic resonance brain studies (the macro scale) are quite adequate, the functioning of a single neuron (the Cajal scale) that receives thousands of inputs, establishes contacts with thousand others and operates animals motor activities remains essentially unknown as well as inadequately studied.

Mammals (including humans), regardless of significant variations, belong to a single vertebrate order that share similar anatomy, analogous musculature, comparable motor activities, and a new type of cerebral cortex (the neocortex). While mammals’ body musculature, limbs anatomy (bones, joints, muscles, and nerves alike) and motor activities have remained essentially unchanged, their motor capabilities have increased through hedgehogs, mice, cats, simians to humans. How a common and shared cerebral cortex with similar type of neurons operates mammals’ increasing motor capabilities is an unresolved question that we are exploring herein.

Prenatal developmental Golgi studies of the motor cortex of hamsters, mice, rats, cats, and humans have provided essential information concerning the unique and shared features of the motor cortex pyramidal neuron among mammals (Marín-Padilla, 1970, 1971, 1978, 1983, 1990, 1992, 1998, 2011, 2012). The prenatal development, morphology, and functional organization of the motor pyramidal neuron are evaluated herein, at the Cajal scale. We are using, preferentially, the human data with additional notes from other mammals.

In the present paper, we are unconcerned with the cortical location of motor activities and/or with the origin of human thoughts, our sole aim is to describe the type of cortical neuron that may capable of operating mammals’ increasing motor capabilities and, in humans, the motor expression of thoughts. A better understanding the human motor cortex pyramidal neuron, at the Cajal level, should provide invaluable and needed information for the correct interpretation of data obtained from the other two -micro and macro- neuronal scales. The query about what make us humans and different from other primates will also be reassessed.

## THE HUMAN BRAIN

What makes us humans, among primates, and capable of language has concerned peoples, from everywhere, since the dawn of our existence as well as philosophers, theologians, writers, and, recently, neuroscientists. Despite innumerable attempts to explain it, the enigma remains unsolved. Most concur that the answer must be in our brain unique structural and functional organizations. Our brain large size and complex structural organization and, more importantly, our capacity for using it for the expression of our mental thoughts and intentions through motor actions are most recent studies essential aims. Perhaps encouraged by the fact that DNA studies (evolution genomics) of primates have failed to provide data for explaining the uniqueness of our brain cognitive activities (Varki and Altheide, 2005; Marques-Bonet et al., 2009).

While some believed that the differences between our brain and that of higher primates are of degree but not of kind (Darwin, 1936) others, unsatisfied with that explanation, continue searching for new answers (Cajal, 1911; Elston, 2003; DeFelipe, 2011; Elston et al., 2011; Marín-Padilla, 2011). Cajal cognizant of the cortex cytoarchitecture pointed out: “It is presumed that the extent and complexity of the gray matter (cerebral cortex) are closely related to the psychological hierarchy of each mammalian species” (Cajal, 1911). De Felipe proposes that the large size of our brain and its great structural complexity have permitted the spectacular development of our cognitive and mental skills (DeFelipe, 2011). The number and density of dendritic spines (synaptic structures) in different cortical areas may reflect functional differences among cortical regions (Elston et al., 2011). Pyramidal cells of the prefrontal cortex have, on average, up to 23 times more dendritic spines than those in the primary visual area (Elston, 2003). The unique structural specialization of pyramidal cells as well as the circuits they establish has permitted the evolution of human cognitive processing to its present state (Elston, 2003). The ongoing specialization of the cortex pyramidal neuron may have played an important role in primate executive cortical functions (Elston et al., 2011). It is interesting that the focus of these studies have been on our cerebrum gray matter (where neurons reside) structural complexity as well as on the nature of its pyramidal neurons, which is explored herein.

These postnatal studies are in the right track since understanding the structure may be the first step for understanding and interpreting function. Cajal celebrated “arrows” are an excellent testimony of that fact. However, how our cerebrum structural complexity is translated into the intricate motor activities involve in language and other unique motor activities remains unsolved. One possible approached for solving this compound riddle might be to reduce it to a developmental study a single neuronal type -the motor pyramidal neuron- that supposedly operates mammals’ motor activities. Cognizant that others neuronal elements do participate and contribute to this neuron functional activity. The pyramidal cell (Cajal’s psychic neuron) is the most abundant and fundamental cell type of mammals’ new cerebral cortex and, in my opinion, a mammalian innovation (Marín-Padilla, 2011). This neuron prenatal development the structural and functional organizations are investigated herein.

## THE MAMMALIAN NEW CEREBRAL CORTEX

Mammals’ new cerebral cortex (neocortex), the last neuronal stratum added to the vertebrate’s brain axis, represents a new type of cortical organization. Its development starts, in the early embryo, as a primordial cortical organization composed of extracortical neurons and afferent fibers and of efferent ones (Marín-Padilla, 1971, 1978, 1983, 2011). In humans, it is first recognized in 6-week-old embryos and is fully established by seven weeks of age. Its formation is a rapid (days) process that extends subpially through the entire brain. It is avascular, precedes the arrival of the new pyramidal neurons and is a prerequisite for their ascending migration and subsequent incorporation (Marín-Padilla, 2011). This primordial cortical organization is also recognized in the early neocortex of mouse, hamster, and cat embryos (Marín-Padilla, 1971, 2011).

Mammals’ primordial cortex cytoarchitecture (neurons, fibers) and superficial plexiform organization resembles the primitive cortex of amphibians and reptiles (Marín-Padilla, 1971, 1998). These similarities are not surprising since amphibian, reptiles, and mammals share similar skeleton, body musculature, four limbs with bones joints muscles and nerves alike as well as analogous early motor activities. Moreover, mammals’ neocortex will have to inherit the original amphibian-reptilian motor blueprints to operate the early embryo’s motor activities. Those motor blueprints are probably incorporated into mammals’ primordial cortical organization. Early mammalian embryos (including humans) share many similar features with other vertebrate’s embryos, including: primitive appearance small heads undulating movements extremities with flippers and a tail. The appearance of a 6-week-old human embryo is not unlike that of any other mammalian embryos. However, by the seventh week of age, the embryo (in only a few days) has become distinctly human due to the extraordinary enlargement of the head frontal region caused by the underlying expanding cerebrum (Marín-Padilla, 1983, 2011). No other mammalian embryo shows this degree of cerebrum and head enlargements.

Mammals’ new cerebral cortex distinguishing features include the combination of a primordial cortical organization and the subsequent incorporation, within it, of a new type of pyramidal neuron.

## THE MAMMALIAN NEOCORTEX NEW PYRAMIDAL NEURON

The neocortex new pyramidal neuron is a mammalian innovation, shared by all, characterized by distinctive developmental, morphological, and functional features. They originate in the cortex ependymal neuroepithelium and attracted by *Reelin* from Cajal–Retzius cells and using radial glial fibers as guides ascend reaching the first layer establishing contacts (dendritic bouquets) and remain functionally anchored to it for life (Marín-Padilla, 1990, 1992). Their incorporation occurs *within* the primordial cortex dividing its components into those above and those below the newly formed and expanding pyramidal cell plate (PCP). Original elements above the plate become the new cerebral cortex first lamina components and those below it components of the so-called subplate zone. In humans, the incorporation of the new pyramidal neurons within the primordial cortex occurs from the 8th to the 15th week of age establishing the neocortex gray matter, where most neurons reside. During this time, all new pyramidal neurons are functionally anchored to first layer by dendritic bouquets, are undifferentiated and their variable sizes reflect their arrival time. The pyramidal-like neurons of amphibians and reptiles primitive cortices share similar functional anchorage to Cajal–Retzius cells and the operation of their motor activities. The human embryo early motor activities are probably operated by the subplate pyramidal-like projective neurons of the primordial cortex since the new pyramidal neurons function does not start until the 15th week of age.

**FIGURE 1:**
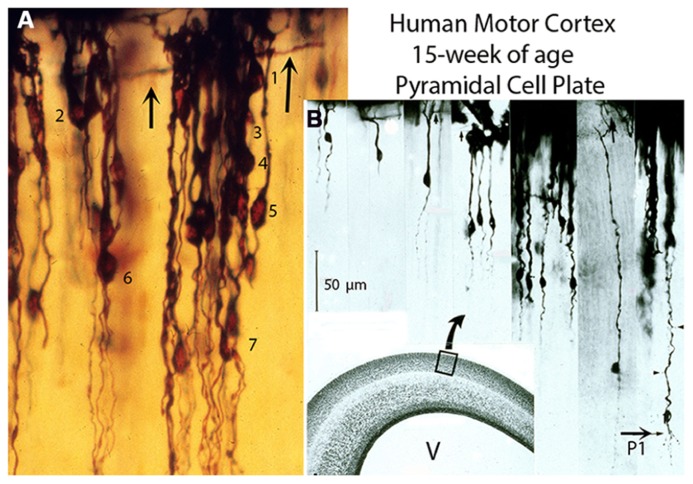
**Composite figure of photomicrographs (A,B) from rapid Golgi preparations of the motor cortex of 15-week-old human fetuses showing the developing gray matter neuronal composition, organization, and stratification**. **(A)** Photomicrograph showing (at high magnification) the developing motor cortex gray matter neuronal composition and distribution through seven (1–7) different strata. At this age, the pyramidal cell plate formation is completed. It is composed only of pyramidal neurons of different sizes, anchored to first lamina, with smooth spineless apical dendrites, bodies without basal dendrites and unbranched descending axons, most of which have no yet reached the underlying white matter. The neuron’s size (apical dendrite length) denotes its arrival time at first lamina. **(B)** Montage of selected photomicrographs of the human motor cortex pyramidal plate showing (at a similar magnification) the neurons different sizes (apical dendrite length) and stratification. Their size ranges from the smaller superficial and last to arrive at first lamina and establish functional contacts (dendritic bouquets) with Cajal–Retzius cells to the larger, deeper and first ones to arrive. The thick horizontal axons (arrows) of Cajal–Retzius cells are also shown **(A,B)**. Scale: 50 μm. Some of the larger, deeper, and older pyramidal neurons have started to develop short basal dendrites (**B**, P1) and a few dendritic spines (arrow heads) indicating the starting functional maturation of the motor cortex first (P1) pyramidal cell functional stratum. The functional maturation of this P1 pyramidal cell stratum is accompanied by the incorporation of its local microvasculature, protoplasmic astrocytes, and inhibitory neurons (Marín-Padilla, 2011). **(B)** Inset. Hematoxylin and eosin (H & E) preparation of the human motor cortex, at 15-week of age, showing the overall composition of its various components: first lamina, gray matter, subplate, white matter, matrix, paraventricular, and ependymal zones and the ventricle (V). (Modified from Marín-Padilla, 2011).

All new pyramidal neurons must ascend, reach the first lamina, develop a dendritic bouquet and become functionally anchored to it (Marín-Padilla, 1990, 1992). Consequently, their apical dendrites, while retaining their original anchorage to first layer, will have to elongate anatomically to accommodate the arrival of subsequent neurons. By the 15th week of age, they have formed a stratified cellular plate about 100 cells thick of closely packed undifferentiated new pyramidal neurons of different sizes all functionally anchored to first layer (**Figures 1A,B**). This cellular plate, sandwiched between first lamina and subplate zone, represents the neocortex gray matter. From the 8th to the 15th week of age, this cellular plate is solely composed of new pyramidal neurons of different sizes with smooth spineless apical dendrites bodies without basal dendrites and unbranched descending axons that start to reach the underlying white matter (**Figures 1A,B**). The apical dendrite length (neuron size) reflects the neuron arrival time at first lamina. The deeper and larger ones were the first to arrive at the first layer, the superficial and smaller ones the last to arrive and the sizes of intermediate ones reflect their variable arrival time (**Figure 1B**). During this developmental stage, the developing gray matter is also crossed by a few ascending white matter afferent fibers without collaterals that reach the first lamina, by ascending radial glial fibers and by descending pial perforating vessels without sprouting capillaries that reach the neocortex lower zones (Marín-Padilla, 2011, 2012). Radial glial fibers bifurcate several times and supply the endfeet needed for the construction and maintenance of the expanding neocortex external glial limiting membrane (EGLM) and for the manufacture of its basal lamina (Marín-Padilla, 1995). The EGLM demarcates anatomically the cerebrum [and central nervous system (CNS)] from surrounding tissues and is only perforated by entering pial capillaries (Marín-Padilla, 2012). In other CNS regions, is also perforated by entering as well as exiting nerves. At this age, the pial perforating vessels descend unbranched through the gray matter reach the subplate, white matter and paraventricular zones and establish anastomotic capillary plexuses among them throughout these zones (Marín-Padilla, 2012).

At the 8th week of age, the apical dendrite length of the deep and older pyramidal neurons is around 15 μm, by the 15th it has anatomically elongated to around 275 μm and at birth it has further elongated, both anatomically and functionally, to about 1,500 μm (**Figure 3A**). The number of spines per apical dendrite has also increased from a few in 15-week-old embryos to around 2,000 in the newborn infant. In cats, the apical dendrite length of deep and older pyramidal neurons increases from 25 μm, in 27-day-old embryos, to about 650 μm by the time of birth and from a few dendritic spines to about 700 (per dendrite) reflecting the developing embryo increasing motor capabilities (Marín-Padilla, 2011).

During the 15th-16th-week of age, the human motor cortex undergoes a series of fundamental structural and functional transformations (Marín-Padilla, 2011). The ascending migration and incorporation of new pyramidal neurons into the developing gray matter is completed. The apical dendrites of the subplate pyramidal-like neurons and the axons of Martinotti cells, both from the primordial cortex, start to loose their original functional contacts with first lamina and undergo a gradual regression (Marín-Padilla, 2011). Eventually, they are transformed into deep (subcortical) interstitial neuron of undetermined function. In the early human embryo the subplate pyramidal-like neurons are likely the source of the projective motor pathway to subcortical centers and eventually to the embryo musculature for controlling his early motor activities.

During the 15th to the 16th week-of age, some of the deepest, older, and larger pyramidal neurons start to develop short basal dendrites a few apical dendritic spines and their descending axons have already reached and penetrated into the underlying white matter (**Figure 1B**). These changes imply the starting functional maturation of the deepest and oldest gray matter neurons and the establishment of the first (P1) pyramidal cell functional stratum (layer V in current nomenclature) in the human motor cortex (**Figure 1B**). These transformations imply that, at this age, the original motor activity of the subplate pyramidal-like neurons ceases while that of the deeper, older, and larger new pyramidal neurons begins. In other words, the embryo’s motor activities operated by the pyramidal-like neurons of the subplate zone are changed to that of the fetus operated by the new pyramidal neurons. The subsequent functional maturation of P1 pyramidal neurons (as well as that of other pyramidal cell strata) will be a progressive, ascending and stratified process, from lower and older to upper and younger strata, induced and operated by the ascending penetration, into the developing gray matter, of thalamic and other afferent fibers from the white matter (**Figure 4A**). Such that while the deeper and older pyramidal neurons have started their functional maturation, at this age, those of the above strata are still undifferentiated.

Furthermore, the gray matter first anastomotic capillary plexus, between contiguous perforating vessels, is also established, at this age, throughout the lower and older pyramidal cells (P1) stratum concomitant with its functional maturation (Marín-Padilla, 2011, 2012). Gray matter protoplasmic astrocytes are also first recognized at this time, only among the gray matter new capillaries (Marín-Padilla, 1995). At this age, transversely migrating undifferentiated cells are also recognized throughout the deepest pyramidal (P1) cell stratum (Marín-Padilla, 2012). These migrating cells, of extracortical origin, represent the precursors of the neocortex inhibitory neurons (Parnavelas, 2007; Marín, 2013). Their arrival coincides with the functional maturation and microvascularization of the gray matter deepest (P1) pyramidal cell functional stratum. At this age, the upper pyramidal cells remain undifferentiated, avascular, without traversing inhibitory neurons and wanting protoplasmic astrocytes.

**FIGURE 2:**
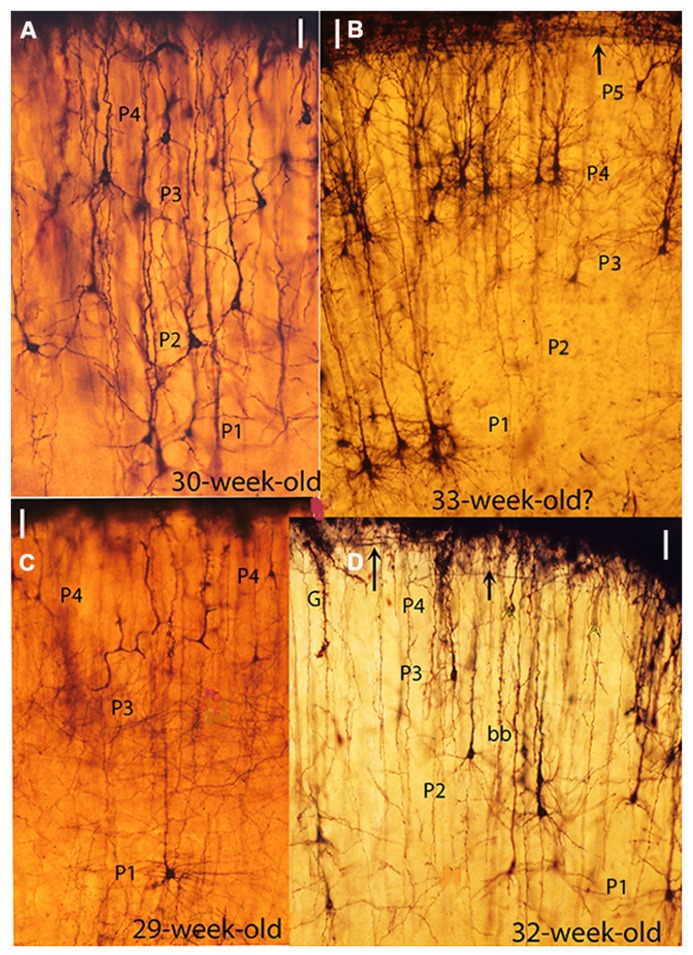
**Composite figure of photomicrographs from rapid Golgi preparations of the motor cortex of human fetuses of 30 (A), 33 **(B)**, 29 **(C),** and 32 **(D)** weeks of age showing the sequential ascending stratification of the gray matter pyramidal neurons functional strata, At this age four basic (P1, P2, P3, and P4) pyramidal cell functional strata are recognized in the motor cortex**. The neurons of each stratum have developed basal dendrites and apical dendritic spines, reflecting their ascending maturation. **(B)** This photomicrograph was obtained from one of Cajal original (1890) Golgi preparations labeled motor cortex of a 33-week-old fetus. However, the presence of a P5 pyramidal cell functional stratum suggests that the fetus was older, possibly 35-week of age. **(D)** Also shows a descending first lamina special astrocyte (G) still attached to the cortex EGLM representing a precursor of gray matter protoplasmic astrocytes as well as a bitufted (bb) neuron. The arrows **(B,D)** mark the presence of Cajal–Retzius thick horizontal axons within the first (I) lamina. The illustrations microscopic magnifications are unequal. (Modified from Marín-Padilla, 2011).

**FIGURE 3:**
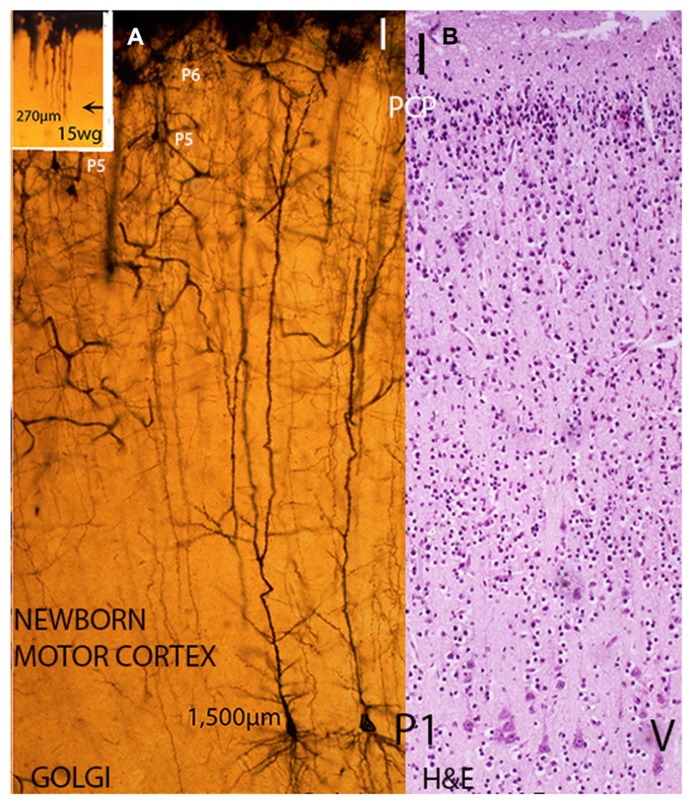
**Composite figure of photomicrographs including a rapid Golgi (A) and a hematoxylin and eosin **(B)** preparation of newborn infants motor cortex showing their different staining capabilities**. While in Golgi preparations **(A)** the whole neuron (dendritic branches with spines and axon with collaterals) is stained as well as the local microvasculature and gray matter protoplasmic astrocytes, in H & E preparations **(B)** only the neurons and glial cells bodies are stained. **(A)** Illustrates, at similar microscopic magnification, the size (apical dendrite length) and dendritic morphology of analogous deep, large, and early motor pyramidal neurons of the P1 stratum from a 15-week-old fetus (inset) and a newborn infant. While remaining functionally anchored to first lamina, the fetus P1 pyramidal neuron size (apical dendrite length) measures about 270 μm, has few short basal dendrites and a couple of dendritic spines (see **Figure 1B**), the newborn motor pyramidal neuron **(A)** has elongated the apical length, both anatomically and functionally to around 1,500 μm. These neurons apical dendrite, the terminal dendritic bouquets within the first lamina, the several collaterals dendrites and the long basal ones all covered by innumerable dendritic spines (postsynaptic structures). Moreover, the pyramidal neuron dendrites will continue to elongate anatomically and functionally during postnatal life while retaining its first lamina functional anchorage and its body cortical location. The cortex motor regions pyramidal neurons will operate the human’s unique motor activities, such as: speaking, writing, painting as well as thinking as a premotor cortical activity. **(B)** H & E preparation of the motor cortex of a newborn infant showing its overall cytoarchitecture, the pyramidal neurons of P1 functional stratum (layer V in current nomenclature), the apparently barren first lamina (I) and a thin remnant of still undifferentiated neurons from the original pyramidal cell plate (PCP) under the first lamina. These neurons will mature functionally during early postnatal life and will incorporate an additional (P7) pyramidal cell stratum of the human motor cortex. (Modified from Marín-Padilla, 2011).

**FIGURE 4:**
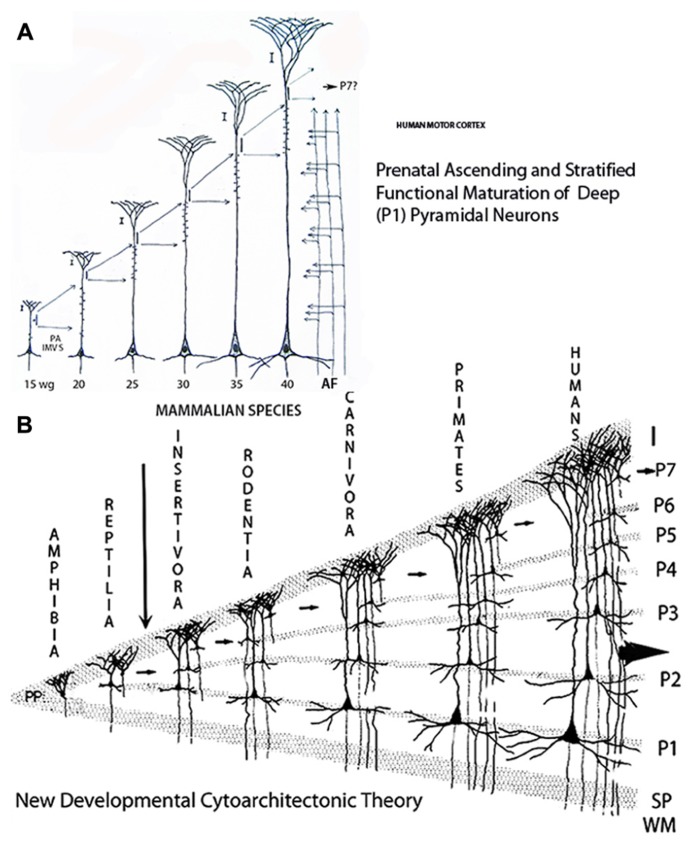
**Composite figure of schematic drawings showing (A) the prenatal sequential, ascending and stratified anatomical and functional maturations of a large, deep, and first to arrive (P1) pyramidal neuron of the human motors cortex; and, **(B)** the vertebrate’s new cerebral cortex evolution and laminar reciprocities through amphibians, reptiles, and mammals**. **(A)** Illustrates a motor pyramidal neuron prenatal development and the anatomical and functional elongations (extensions) of its apical dendrite retaining its original functional attachment to first lamina and the location (cortical depth) of its body. During each developmental stage, the neuron incorporates an additional segment of functional (synaptic) membrane to its apical dendrite induced and regulated by the ascending penetration of thalamic and other afferent fibers from the white matter. The human motor pyramidal neurons, after completing their ascending migration (from the 8th to the 15th week of age), incorporate the first functional segment at 15-week of age, a second functional segment is incorporated at 20-week of age, a third one at 25, a fourth one at 30, a fifth one at 35, and a sixth one by birth time. A remnant of the original undifferentiated pyramidal cell plate, shared by all newborn mammals, will mature during early postnatal life and incorporates an additional functional segment to the pyramidal neuron. **(B)** Schematic drawing illustrating the proposed developmental cytoarchitectonic theory concerning the ascending functional maturation and stratification (evolution) of vertebrates cerebral cortex and the laminar (strata) correspondences among amphibians, reptiles and mammals. The small arrows indicate the undifferentiated pyramidal cell remnant participation in the evolution of the additional pyramidal cell functional strata in the course of mammalian evolution. The large arrow indicates the human cerebrum evolving possibilities as an open biological system capable of further stratification and progression. The drawing has no other implications concerning mammals’ evolution that certainly is not a lineal one (Modified from Marín-Padilla, 2011).

**FIGURE 5:**
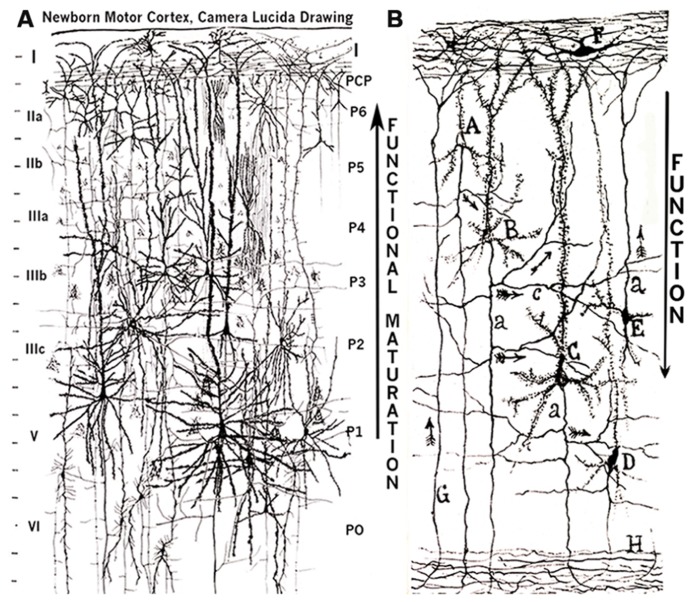
**Composite figure of camera lucida drawings from rapid Golgi preparations illustrating (A) the newborn infant motor cortex overall neuronal composition, stratification, and ascending functional maturation (large ascending arrow) from lower and older to upper and younger pyramidal cell strata and **(B)** an original Cajal drawing illustrating the cerebral cortex descending cascade of functional inputs (arrows) from upper (younger) to lower (older) pyramidal cell strata**. **(A)** This drawing illustrates (at similar magnification) the various pyramidal cells functional strata from the deeper older and larger neurons to the superficial smaller and younger ones with several intermediate ones, various type of inhibitory (baskets and bi-tufted, chandeliers) neurons, Martinotti cells with ascending axons and first lamina with Cajal– Retzius cells and long horizontal axons and the terminal dendritic bouquets if all underlying pyramidal neurons. The drawing also illustrates the correspondences between the current descending (layers I, II, III, IV, V, and VI) laminar nomenclature (left side) with the proposed ascending one (P1, P2, P3, P4, P5, P6) supported by developmental data (right side). A thin remnant of the original pyramidal cell plate (PCP) is also illustrated under the first lamina. **(B)** Cajal’s original drawing illustrating his conception of the cerebral cortex descending functional activity. Also illustrated are first lamina Cajal–Retzius cell (F), the numerous horizontal axonic fibers and the terminal dendritic bouquets of the underlying pyramidal neurons. The first lamina also receives (ascending small arrows) the terminal of afferent fibers (G) from the white matter and the axons of Martinotti (E) cells. The cortex descending functional activity results from the combination of the interconnecting and descending functional inputs (small arrows) from the upper (A) through the intermediate (B,C) to the deeper (D) pyramidal cell strata. The pyramidal neurons descending axons also reach the white (H) matter. The deeper pyramidal neurons that receive cascading functional inputs from all upper pyramidal neurons are the source of the projective motor pathways to subcortical centers and eventually to the animal musculature. I have incorporated to two large arrows: an ascending (functional maturation) one to my drawing and a descending (function) one to Cajal’s.

The gray matter ascending functional maturation involve the pyramidal neurons of each subsequent stratum and will include the local incorporations of inhibitory neurons microvasculature and protoplasmic astrocytes on each ascending strata (**Figures 1–5**). The gray matter ascending neuronal, microvascular, and glial maturations increases the neocortex thickness (upwardly) as well as the anatomical and functional elongations of all pyramidal neurons’ apical dendrites. The neocortex eventual thickness should reflect the pyramidal neurons eventual sizes (apical dendrite length) as well as the number of pyramidal cell functional strata established on each mammalian species cerebrum (**Figure 4B**).

From the 15th-week of age to birth and postnatally, the new pyramidal neurons apical dendrites continue to elongate functionally by the incorporation of additional synaptic membrane, while retaining their original functional anchorage to first layer (**Figures 2**, **3**, **4A**, and **5A**). The development of basal dendrites and the appearance of apical dendritic spines have been the criteria for determining the starting functional maturation of the new pyramidal neurons of each ascending stratum. The human motor cortex gray matter functional maturation is an ascending and stratified process that starts around the 16th week of age with the establishment of the first (P1) pyramidal cell functional stratum, layer V in current nomenclature (**Figure 1B**). The motor cortex subsequent ascending and stratified functional maturation will progress throughout late prenatal and early postnatal life (Marín-Padilla, 2011). By the 20th week of age a second (P2) pyramidal cell functional stratum is added above the first one and, consequently, the apical dendrite of P1 pyramidal neurons will further elongate, both anatomically and functionally. This dual developmental process: the incorporation of a new pyramidal cell functional stratum and the elongation of the apical dendrite of all pyramidal neurons of previous strata will be repeated during the cortex prenatal functional maturation (Marín-Padilla, 1992, 2011). By the 25th a third (P3) pyramidal cell functional stratum is added above the previous ones; by the 30th a fourth (P4) pyramidal cell stratum is added; by the 35th a fifth (P5) one is added and by the 40th a sixth (P6) one start to mature (**Figures 1–5**). The pyramidal neurons ascending and stratified functional maturation is accompanied by the concomitant incorporation, on each new stratum, of inhibitory neurons, microvasculature, and protoplasmic astrocytes (Marín-Padilla, 2011). Consequently, the first (P1) pyramidal cell functional stratum (layer V in current nomenclature) will be shared by all mammals and will control the developing fetus motor activities (**Figure 4B**). These neurons axons become the source of the main projective motor pathways to subcortical centers and eventually to the animal musculature, for life (**Figure 4B**). The number of pyramidal cell functional strata in the cerebrum of each mammalian species reflects and parallels its motor proficiencies and abilities and increases during their evolution (**Figure 4B**). This figure simply reflects this paper basic conception without any additional implication.

Furthermore, the motor cortex of newborn mammals, I have studied, still have a thin remnant, under the first lamina, of the original undifferentiated PCP (**Figure 3B**). These neurons will mature functionally during the animal’s postnatal life and will incorporate an additional pyramidal cell functional stratum to each species motor cortex (**Figure 4B**, small arrows). Their postnatal maturation would help, each mammal, to confront new environmental challenges and to develop novel and appropriate motor activities to maneuver them. It would seem that each mammalian species develop the number of pyramidal cell functional strata needed for operating its motor activities and for confronting new challenges during their postnatal existence (**Figure 4B**). Moreover, I have recognized remnants of undifferentiated pyramidal cells, under the first lamina, even in the motor cortex of some adult mammals. Perhaps, suggesting the possibility of developing new motor capabilities throughout the mammal’s postnatal life. The possible significance of this feature should be further investigated in postnatal brains studies.

The human newborn motor cortex is also characterized by the presence, under first lamina, of a thin remnant of the original undifferentiated PCP (**Figures 3A,B**, **4A,B**, and **5A**). This remnant matures postnatally and is no longer recognizable in the motor cortex of young children. Its postnatal functional maturation adds an additional (P7) pyramidal cell stratum to the human motor cortex (**Figures 4B** and **5A,B**). The presence of both P6 and P7 pyramidal cell strata distinguishes the human motor cortex from that of other primates. The functional activity of these additional P6 and P7 pyramidal cell strata should participate in the operation of our unique motor and mental capabilities including language as well as the capacity of thinking as a premotor activity.

The new pyramidal neuron essential and distinguishing feature is the capacity of elongating – anatomically and functionally – its apical dendrite and of incorporating additional sensory information without losing its original anchorage to first layer or the location (cortical depth) of its soma. Actually, without altering its essential nature throughout the course of mammalian evolution (Marín-Padilla, 1992). By increasing its main receptive surface (apical dendrite and collaterals), the new pyramidal neuron can incorporate the additional sensory information needed for controlling mammals’ increasing motor activities (**Figures 1–5**). Such that the length of the apical dendrite (neuron size), the amount of synaptic information it could store (dendritic spines and other synaptic contacts) and the number of functional strata should reflect and distinguish each mammalian species cerebrum motor capabilities. Comparatively, the apical dendrite length of deep, large, and old pyramidal neurons of the mouse neocortex should be shorter and should storage less amount of synaptic information than those of a human and those of cats should be about intermediate (Marín-Padilla, 1967, 2011; Valverde, 1967). In the three instances, the new pyramidal neuron essential nature has remained unaffected while its size (apical dendrite length) and motor competence have increased. In my opinion, any neuron that does not establish original contacts with first lamina and/or lose them, despite the shape of its soma, should not be considered and/or labeled, as mammalian pyramidal neuron.

The prenatal development of pyramidal neurons in other mammalian species should be studied because the available data is deficient.

## A NEW CYTOARCHITECTONIC THEORY

Based on these developmental observations new developmental cytoarchitectonic theory has been proposed for the evolving mammalian neocortex (Marín-Padilla, 1992). The new theory proposes that the mammalian neocortex development is an ascending and stratified processes and that the number of pyramidal cells functional strata (laminae) increases paralleling the mammals’ increasing motor capability (**Figure 4B**). Accordingly, the hedgehog motor cortex essentially requires only two pyramidal cell functional strata to accomplish all its motor needs, the mouse 3, the cat 4, the monkey 5, and humans 6 and a seventh one added postnatally (**Figure 4B**). In addition, the newborn motor cortex in all mammalian species still has an undifferentiated pyramidal cell stratum, under first lamina, that will mature postnatally adding an additional stratum to the motor cortex. The postnatal functional maturation of this last stratum prepares the animal for confronting and operating new challenges by novel motor activities (**Figure 4B**, small arrows). The progressive ascending penetration of white matter thalamic and other afferent fibers into the developing gray matter induces and regulates the gray matter ascending and stratified functional maturation. As new motor activities evolve during mammalian evolution the penetration of afferent fibers (from thalamic and cortical sources) continues to ascend (penetrate) into the developing gray matter inducing the functional maturation of the additional pyramidal cell strata that characterize each new species. The number of neocortical neurons (genetically determined) that characterizes each mammalian species has also increased during their evolution. There seen to be a tendency for the width of mammals’ neocortex to increase in the direction of highly organized motor activities (Blinkov and Glezer, 1968).

## NEOCORTEX DESCENDING VERSUS ASCENDING STRATIFICATION

The current understanding of the mammalian neocortex lamination (stratification) needs to be reevaluated as two opposite conceptions: the descending one currently used and the ascending one suggested herein, have been proposed (**Figure 5A**). The classic and universally accepted theory proposes that the mammalian neocortex is subdivided into a series of descending laminae (layers I, II, III, IV, V, and VI). Although unsupported by developmental data, this old theory (Broadman, 1909) has been universally accepted without any challenge and/or validation. Moreover, the idea that the mammalian neocortex, from edentates to primates, has (essentially) six descending laminations (strata) is arbitrary and unsupported by developmental data. To maintain the six cortical laminae (strata) for all mammalian species has lead to using arbitrary nomenclatures, including laminar concentrations for lower mammalian species (lamina II–III–IV for edentates and lamina II–III for rodents) as well as laminar duplications (laminae IIa and IIb) for primates and even triplications (laminae IIIa, IIIb, and IIIc) for humans (Marín-Padilla, 1978). Reductions as well as duplications of neocortical laminae are arbitrary and fail to reflect the neocortex development, neurohistology, and functional activity as well as mammals increasing motor capabilities. In the course of mammalian evolution, the number of pyramidal cell functional strata (laminae) has actually increased to reflect their increasing motor capabilities (**Figure 4B**).

This dual conundrum needs also to be resolved because of the obvious and significant functional implications involve. It is essential to establish if the neocortex cytoarchitecture is a descending and/or an ascending process. Likewise, the number laminations (strata) in mammals’ neocortex need to be established. Such clarifications are beyond the scope of the present study.

## ASCENDING MATURATION VERSUS DESCENDING FUNCTION

The functional maturation of the neocortex gray matter (pyramidal, non-pyramidal and inhibitory neurons, blood capillaries, protoplasmic astrocytes, and penetration of afferent fibers) is an ascending and stratified process from lower and older strata to superficial and younger strata. However, the neocortex functional activity is a descending process as originally proposed by Cajal (1933) and corroborated by recent neurophysiologic studies (Weiler et al., 2008).

The deepest P1 pyramidal neurons, shared by all mammals, are the essential projective elements to subcortical centers and eventually to the animal musculature. These projective neurons receive a descending functional cascade from all pyramidal neurons of the upper strata (**Figure 5B**). The axons of upper pyramidal neurons establish functional contact with the dendrites of lower strata neurons establishing a descending functional cascade from upper and recent strata to lower and older ones (**Figure 5B**). Inhibitory as well as non-pyramidal neurons of each stratum also participate and regulate this cascading functional activity. For each mammalian species, the amount of information received by the deepest, older and projective P1 pyramidal neurons will be a combination of inputs received from all pyramidal neurons of the above strata. Their functional output to subcortical centers and eventually to the animal musculature will be selected from this descending functional cascade. The operating capacity and the complexity of the descending functional cascade upon mammals’ shared musculature will depend on the number of participating pyramidal cell strata. The greater the number of pyramidal cell functional strata the greater the mammal’s motor capabilities. The fact that the number of pyramidal cell functional strata as well as mammal’s motor capabilities have concomitantly increased in the course of mammalian evolution will further corroborate these assumptions (Marín-Padilla, 1978, 1992).

## CONCLUSION

While mammals share similar body anatomy, four extremities, analogous musculatures, and motor activities their motor capabilities have progressively increased in the course of their evolution. How mammals operate their increasing motor capabilities using common and shared skeletal, muscular and nervous structures remain unexplained. A possible developmental explanation, supported by prenatal Golgi studies of the human motor, is offered herein. All mammals share a new cerebral cortex (neocortex) and a new type of pyramidal neuron that represent mammalian innovations. Mammals’ new pyramidal neuron distinguishing feature is the capacity of elongating its apical dendrite (main receptive surface), both anatomically and functionally, without losing its essential nature. In other words, without losing its original functional anchored to first lamina or the cortical depth of its body. By increasing its main receptive surface (apical dendrite and its collaterals), this new type of neuron may be capable of incorporating the additional sensory information needed for operating mammals’ increasing motor capabilities without altering its essential nature. Moreover, the motor cortex of all newborn mammals, I have studied, has a thin remnant of the original undifferentiated PCP under the first lamina. These neurons will mature during postnatal life and incorporate an additional pyramidal cell functional stratum into each mammalian species motor cortex. The functional activity of this additional pyramidal cell stratum should prepare them, during postnatal life, for confronting new environmental challenges as well as for developing novel and appropriate motor activities to manage and operate them. Each mammalian species develops the number of pyramidal cell functional strata needed and necessary for operating its motor activities. Consequently, the number of pyramidal cell functional strata in the neocortex has increased concomitantly with the animal increasing motor capabilities. Non-pyramidal and inhibitory neurons, blood vessels and glial cells are also sequentially and concomitantly incorporated into these ascending functional strata and will co-participate in their functional activity. These simple evolutionary strategies, development of a new cerebral cortex and of a new type of pyramidal neuron, shared by all mammals, operate their increasing motor capabilities by reusing essentially analogous body parts, musculature, extremities, cerebral structures, and neural parkways.

The presence of additional P6 and P7 pyramidal cell functional strata distinguishes the human cerebrum. The presence in our cerebrum of these two additional pyramidal cell strata as well as the learned (not inherited) capability of using them effectively is what distinguished us from other primates. Possibly, the learned functional activity of these additional strata operates our species unique cognitive capabilities, the motor expression of mental thoughts through the use of language as well as through other uniquely human motor activities, such as writing, painting, sculpturing, making and playing music, and practicing sports. The participation of other cortical areas (frontal, visual, parietal, and temporal) is certainly implanted in our cognitive activities. The entire brain (a premotor organ) participates in our cognition but the motor activities are channeled through the motor region pyramidal neurons and eventually to musculature. Words expressing my thoughts are readily understood by other humans, become incorporated into their mental cognition and their ensuing thoughts are translated into words that I could also readily understand. This simple interchange of human thoughts through motor activities has existed since the dawn of our existence. It represents a simply motor activity, wanting any mystery, performed by the functional activity of our cerebrum new pyramidal neurons. There are no limits to our learned capability for expressing thoughts and/or intentions through motor actions.

Essentially, our cerebrum is a premotor organ that commands and operates all our motor activities, including language, and what distinguishes us as a unique species. Homo sapiens represent a new and unique evolutionary stage of primates that, in my opinion, has humanized his primate brain by incorporating additional motor capabilities. The Homo sapiens present evolutionary stage is not a final one as mammal’s new cerebral cortex is an open biological system capable of evolving further by incorporating additional pyramidal cell functional strata. Perhaps, Homo sapiens have the potential of evolving further into a more humane species (Homo humanus) by educating his brain and himself and by improving his conduct toward others. Although this possible evolutionary step has occurred but only in a very few and isolated instances throughout his existence. In my opinion, human are not born, they (he and she) have to make themselves by operating through the cerebrum the motor neurons of the sixth and seventh pyramidal cell strata, unique to the species, for thinking (premotor activity) and expressing their thoughts through motor actions.

The developmental information and ideas outlined herein are offered are proposals based on rapid Golgi observations of the prenatal development of the human motor cortex pyramidal neurons. They have emphasized that the new cerebral cortex (neocortex) and the new type of pyramidal neuron are mammalian innovations. Also that mammals’ new pyramidal neuron is capable of elongating, anatomically and functionally, its apical dendrite and incorporate the additional sensory information needed for operating their increasing motor capabilities. From edentates, to rodents to carnivores to primates to humans, this unique and shared type of pyramidal neuron has progressively extended anatomically and functionally its apical dendrite increasing its receptive surface and motor capabilities without losing its essential nature. The evolution of the human brain evolution is not yet final as new obstacles (extraterrestrial explorations) will arise that will require his attention, study and resolution through new motor activities.

I would like to emphasize that this paper simply introduces a hypothesis concerning the development of mammals’ new pyramidal neurons and has no additional pretentions and/or goals. It is based on prenatal developmental Golgi studies of the human motor cortex pyramidal neurons and of its associated neuronal, microvascular, and glial systems. To corroborate, validate and/or invalidate the structural and functional concepts concerning mammals’ new cerebral cortex and new type of pyramidal neuron introduced in this paper, additional inquiries and studies will certainly be needed.

## Conflict of Interest Statement

The author declares that the research was conducted in the absence of any commercial or financial relationships that could be construed as a potential conflict of interest.

## References

[B1] Blinkov S. M. Glezer I. I. (1968). *The Human Brain in Figures and Tables.* New York: Plenum Press

[B2] Broadman B. (1909). *Verleichende Lokalisarionslehre der Grosshirnrinde in ihren Prizipiens Dargestellt auf grund des zellebauses*. Leipzig: Barth.

[B3] Cajal S. y R. (1911). *Histologie du systéme nerveux de l’homme et des vertebrés.* Paris: Maloine (Reprinted by CSIC, Madrid, 1952)

[B4] Cajal S. y R. (1933). “¿Neuronismo o reticularismo? Las pruebas objetivas de la unidad anatómica de las células nerviosas,” in *Archives de Neurología,* Vol.13 (Madrid: Cajal Institute) 58–104 (Reprinted by the Cajal Institute, Madrid, 1952)

[B5] Darwin C. (1936). *The Origin of the Species and the Descent of Man*. New York: The Modern Library

[B6] DeFelipe J. (2011). The evolution of the brain, the human nature of cortical circuits, and intellectual creativity. *Front. Neuroanat.* 5: 29 10.3389/fnana.2011.00029 PMC3098448 21647212

[B7] Elston G. N. (2003). Cortex, Cognition and the cell: new insights into the pyramidal neuron and prefrontal function. *Cereb. Cortex* 13 1124–1138 10.1093/cercor/bhg093 14576205

[B8] Elston G. N. Benavides-Piccini R. Elston A. Rimange P. DeFelipe J. (2011). Pyramidal cell in prefrontal cortex of primates: marked differences in neuronal structure among species. *Front. Neuroanat.* 5: 2. 10.3389/fnana.2011.00002 PMC3039119 21347276

[B9] Llinás, R. R. (2001). *I of the Cortex (From Neurons to Self)*. Cambridge: MIT Press

[B10] Marín O. (2013). Cellular and molecular mechanisms controlling the migration of neocortical interneurons. *Eur. J. Neurosci.* 38 2019–2029 10.1111/ejn.12225 23651101

[B11] Marín-Padilla M. (1967). Number and distribution of the apical dendritic spines of the layer V pyramidal neurons in man. *J. Comp. Neurol.* 131 465–490 10.1002/cne.901310407 5584088

[B12] Marín-Padilla M. (1970). Prenatal and early postnatal ontogenesis of the human motor cortex. A Golgi study. I. The sequential development of the cortical layers. *Brain Res.* 23 167–183 10.1016/0006-8993(70)90037-5 4097697

[B13] Marín-Padilla M. (1971). Early prenatal ontogenesis of the cerebral cortex (neocortex) of the cat (*Felis domestica*). A Golgi study. Part, I. The primordial neocortical organization. *Z Anat. Entzwickl-Gesch.* 134 117–145 10.1007/BF00519296 4932608

[B14] Marín-Padilla M. (1978). Dual origin of the mammalian neocortex and evolution of the cortical plate. *Anat. Embryol.* 152 109–126 10.1007/BF00315920 637312

[B15] Marín-Padilla M. (1983). Structural organization of the human cerebral cortex prior to the appearance of the cortical plate. *Anat. Embryol.* 168 21–40 10.1007/BF00305396 6650855

[B16] Marín-Padilla M. (1990). Three-dimensional structural organization of layer I of the human cerebral cortex: a Golgi study. *J. Comp. Neurol.* 229 89–105 10.1002/cne.902990107 2212113

[B17] Marín-Padilla M. (1992). Ontogenesis of the pyramidal cell of the mammalian neocortex and developmental cytoarchitectonics: a unifying theory. *J. Comp. Neurol.* 321 223–240 10.1002/cne.903210205 1500541

[B18] Marín-Padilla M. (1995). Prenatal development of fibrous (white matter), protoplasmic (gray matter), and layer I astrocytes in the human cerebral cortex: a Golgi study. *J. Comp. Neurol.* 358 1–19 10.1002/cne.903570407 7545703

[B19] Marín-Padilla M. (1998). Cajal–Retzius cell and the development of the neocortex. *Trends Neurosci.* 21 64–71 10.1016/S0166-2236(97)01164-8 9498301

[B20] Marín-Padilla M. (2011). *The Human Brain: Prenatal Development and Structure*. Heidelberg: Springer 10.1007/978-3-642-14724-1

[B21] Marín-Padilla M. (2012). The human brain intracerebral microvascular system: development and structure. *Front. Neuroanat.* 6: 38 10.3389/fnana.2012.00038 PMC3440694 22993505

[B22] Marques-Bonet T. Ryder O. A. Eichler E. E. (2009). Sequencing primate genomes: what have we learned? *Annu. Rev. Genomics Hum. Genet.* 10 355–386 10.1146/annurev.genom.9.081307.164420 19630567 PMC6662594

[B23] Ortega y Gasset J. (1957). *El Hombre y la Gente. Revista de Occidente*. Madrid: Alianza Editorial.

[B24] Parnavelas J. G. (2007). Molecular mechanisms involved in the migration of cortical interneurons. *J. Anat.* 210 605–605 10.1042/AN20090053 16519654

[B25] Sperry R. (1952). Neurology and the mind-body problem. *Am. Sci.* 40 291–312

[B26] Valverde F. (1967). Apical dendritic spines of the visual cortex and light deprivation in the mouse. *Exp. Brain Res.* 3 337–352 10.1007/BF00237559 6031165

[B27] Varki A. Altheide T. K. (2005). Comparing the human and chimpanzee genomes: searching for needles in a haystack (Invited Perspective). *Genome Res.* 15 1746–1758 10.1101/gr.3737405 16339373

[B28] Weiler N. Wood L. Yu J. Solla S. A Shepherd G. M. G. (2008). To-down laminar organization of the excitatory network in the motor cortex. *Nat. Neurosci.* 11 360–366 10.1038/nn2049 18246064 PMC2748826

[B29] Wolpert D. M. Flanagan J. R. (2010). Motor Learning. *Curr. Biol.* 20 467–472 10.1016/j.cub.2010.04.035 20541489

